# Antibacterial activity of two newly isolated *Bdellovibrio bacteriovorus* strains on *Salmonella enterica* serovars of food safety concern

**DOI:** 10.1128/spectrum.00861-25

**Published:** 2025-09-15

**Authors:** Yewande O. Ajao, Lari M. Hiott, Laura E. Williams, Charlene R. Jackson, Jonathan G. Frye

**Affiliations:** 1 Poultry Microbiological Safety and Processing Research Unit, Agricultural Research Service, US Department of Agriculture, Athens, Georgia, USA; 2 Oak Ridge Institute for Science and Education 17215, Oak Ridge, Tennessee, USA; 3 Georgia Institute of Technology 1372, Atlanta, Georgia, USA; University of Georgia College of Veterinary Medicine, Athens, Georgia, USA

**Keywords:** predatory bacteria, *Bdellovibrio bacteriovorus*, *Salmonella enterica*, periplasmic predators, biological control

## Abstract

*Bdellovibrio* are obligate predators and have been described as living antibiotics since their predatory lifestyle enables them to kill pathogenic bacteria, making *Bdellovibrio* a promising biological control agent. *Bdellovibrio* strains were isolated from sampling sites in an urban watershed and tested for their killing activity on *Salmonella enterica* strains associated with human infections. An enrichment technique was used to isolate *Bdellovibrio* from surface water samples from local tributaries to the Oconee River, employing *Salmonella* Infantis as the prey bait. The isolated *Bdellovibrio* strains were sequenced; their prey range activity was tested against a panel of clinically significant *S. enterica* serovars, while predation efficiency was tested on *S*. Infantis. The result demonstrated the ability of two newly isolated periplasmic *Bdellovibrio bacteriovorus* strains, *B. bacteriovorus* YOA24 and *B. bacteriovorus* YOA38, to kill 11 antibiotic-resistant *S. enterica* serovars and effectively reduce *S*. Infantis populations. The strains were identified as periplasmic *Bdellovibrio* and members of *B. bacteriovorus* based on phenotypic, microscopic, and genotypic characterization. *B. bacteriovorus* YOA 24 and YOA38 demonstrated the ability to reduce *S*. Infantis by 2 logs in 24 h. The killing activity of *B. bacteriovorus* YOA24 and YOA38 indicates their potential to manage *Salmonella* outbreaks. Therefore, *B. bacteriovorus* strains YOA24 and YOA38 can be considered for development as therapeutic agents or probiotics against *Salmonella*.

**IMPORTANCE:**

*Bdellovibrio* is the most studied obligate predatory bacteria. It has potential for use as biological control of gram-negative bacteria in health, agriculture, and the food industry. Most basic research and applications use the type strain *Bdellovibrio bacteriovorus* HD100 or a closely related strain 109J. Screening for other *Bdellovibrio* strains and their killing activity should be explored, knowing that prey range and efficiency could differ among *Bdellovibrio* strains. This study presents two newly isolated *B. bacteriovorus* strains, YOA24 and YOA38, with lytic activity on *Salmonella enterica* serovars. *B. bacteriovorus* YOA24 and YOA38 represent a biological control agent for foodborne *S. enterica* serovars due to their killing activity on the important *Salmonella* strains tested.

## INTRODUCTION

The health challenges associated with gram-negative bacterial infections and disease (1, 2), their continuous increase in antibiotic resistance, and the economic loss attributed to these pathogens (1, 3) are burdensome and call for suitable and effective biological control agents. Predatory bacteria such as *Bdellovibrio bacteriovorus* have been suggested as potential biological control agents of gram-negative antibiotic-resistant bacteria (4, 5).

*B. bacteriovorus* are obligate predators that kill gram-negative bacteria and remove bacterial biofilms (6, 7). Their predation strategy involves attaching to the prey cell, moving into the periplasmic space of the prey, using enzymes to break down prey constituents, using these for growth and division into progeny, and leaving the dead prey cell to start another life cycle (8). The predatory lifestyle of *Bdellovibrio* and their large repertoire of lytic enzymes make them attractive as biological control agents (9). *Bdellovibrio*’s targeted prey includes human and animal pathogens, multidrug-resistant bacteria, and emerging pathogens of health concern, including the zoonotic and foodborne pathogen *Salmonella*.

The application of *Bdellovibrio* on various *Salmonella* serotypes, including antibiotic-resistant strains, is worth exploring. While the prey’s antibiotic resistance profile does not affect susceptibility to *Bdellovibrio* predation, the prey’s outer membrane could influence their susceptibility (5, 10). *B. bacteriovorus* has been reported to have a broad prey range compared to bacteriophages and has been described as a versatile predator (11). However, prey preference has been observed among *Bdellovibrio* strains. The predatory strains reported in most prey range studies have been *B. bacteriovorus* HD100, the type strain for members of this species, and a closely related strain, *B. bacteriovorus* 109J, the most well-described laboratory strain. Considering that *Bdellovibrio* is ubiquitous, each strain has its prey range and efficiency. Other naturally occurring *Bdellovibrio* strains should be characterized for their predatory activity.

Thus, this study aimed to isolate *Bdellovibrio* from natural environments and assess their prey range and predation efficiency against *Salmonella enterica* serovars associated with foodborne diseases. We report the isolation of two *B. bacteriovorus* strains, their relationship to *B. bacteriovorus* HD100 and 109J, and their predatory activity against *S. enterica* serovars with varying phenotypic and genotypic characteristics.

## RESULTS

### *Bdellovibrio bacteriovorus,* capable of killing S*almonella,* are members of the microbial communities in an urban watershed

**Fig 1:**
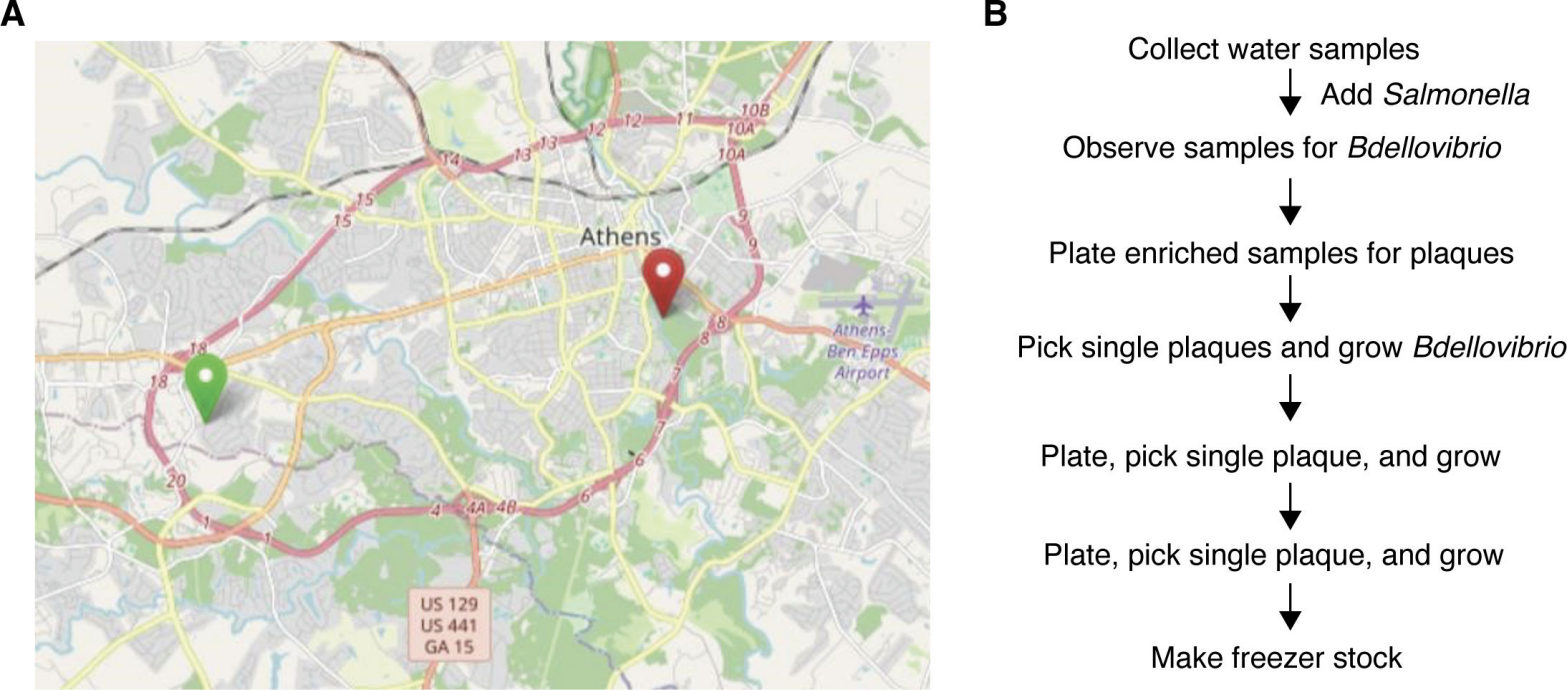
(**A**) Locations from which the *B. bacteriovorus* strains were isolated. Water samples were collected on 18 August 2023. YOA24 was isolated from water collected at the westernmost location (green marker), and YOA38 was isolated from water collected at the easternmost location (red marker). (**B**) A simple workflow for isolating the bacterial predators presented in this study. Map data from OpenStreetMap.

Two *Bdellovibrio* strains, *B. bacteriovorus* YOA24 and YOA38, were isolated from two surface water sites in Athens, Georgia, using *Salmonella* Infantis FSIS32003110 pESI as the prey bait (Fig. 1A). A simple workflow illustrating the isolation protocols for strains YOA24 and YOA38 is represented in Fig. 1B. *S*. Infantis FSIS32003110 pESI was used as bait in the *Bdellovibrio* isolation protocol because this *Salmonella* strain has been associated with several outbreaks of human diseases attributed to poultry contamination. The collected sample was enriched with *S*. Infantis pESI to select *Bdellovibrio* capable of killing this outbreak-associated strain, thereby recovering *Bdellovibrio* strains ideal for applications to control foodborne *Salmonella*.

**Fig 2:**
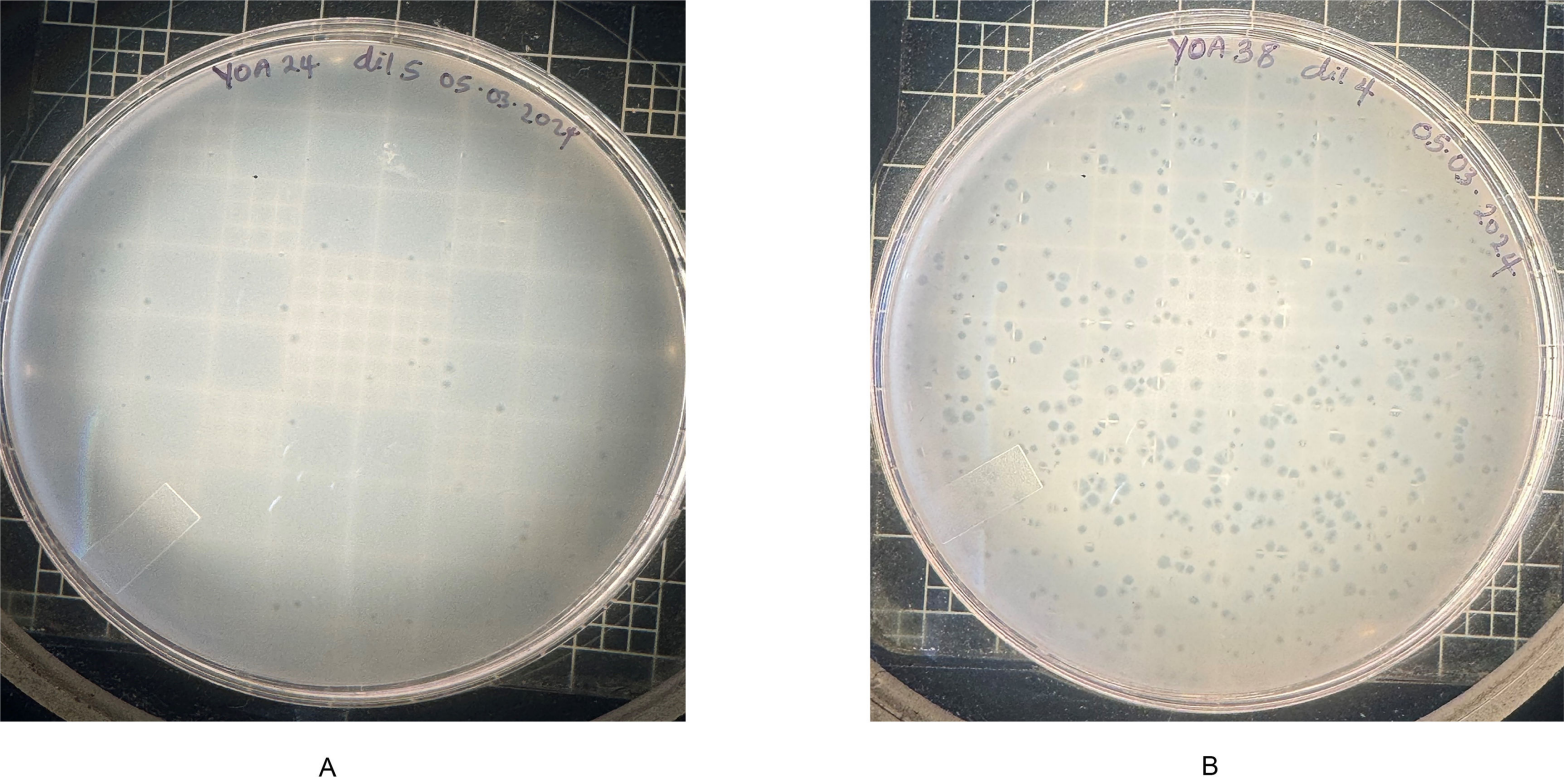
(**A**) and (**B**) show purified *B. bacteriovorus* YOA24 and YOA38 plaques on diluted nutrient broth agar using *S*. Infantis as the prey.

At 48 h, the enrichment for each sampling site showed fast-moving, tiny bacteria typical of *Bdellovibrio*. Under the phase-contrast microscope, these bacteria could attach to *S*. Infantis FSIS32003110 pESI, transforming the *Salmonella* cells into a rounded shape—an attack strategy typical of obligate periplasmic *Bdellovibrio*. The enrichments showing evidence of *Bdellovibrio* were plated in modified HEPES (HM) agar using the double-overlay technique to recover plaque-forming bacteria, using the same *Salmonella* strain for the prey lawn. To obtain pure cultures of these predatory bacteria, three rounds of plaque picking were performed, yielding two isolates designated YOA24 and YOA38. YOA24 and YOA38 on HM agar plates appear as plaque-forming bacteria at 72 h (Fig. 2). These isolates were identified as members of *B. bacteriovorus* based on whole-genome sequencing data.

### YOA24 and YOA38 are phylogenetically related to *B. bacteriovorus* HD100

The assembled genome statistics of YOA24 (GCF_048286975.1) and YOA38 (GCF_048286055.1) are presented in Table S1. BLAST analysis of the 1,514 bp 16S rRNA gene sequence recovered from the genomes of YOA24 and YOA38 to the rRNA type strains/16S ribosomal RNA database showed that strains YOA24 and YOA38 have 99.85% identity with *B. bacteriovorus* HD100 (accession number NR_027553.1). A BLAST of the same sequence on the EZBioCloud 16S-based ID identified YOA24 and YOA38 as members of the *B. bacteriovorus* with a similarity of 99.72% to HD100 (accession number BX842601). A percentage similarity of >98.5% with the type strain HD100 placed YOA24 and YOA38 in the same species as the type strain. In addition, the >70% dDDH (digital DNA-DNA hybridization) with the type strain HD100 confirmed that YOA24 and YOA38 belong to the *B. bacteriovorus*.

**Fig 3:**
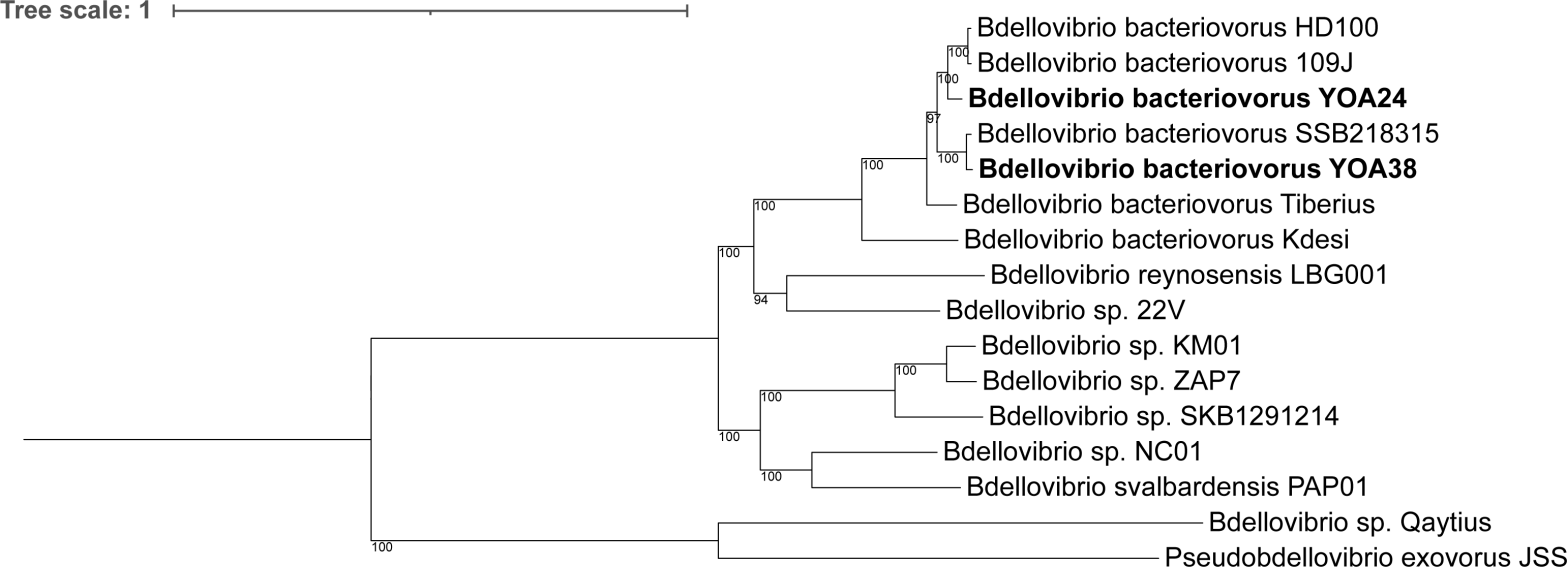
A whole-genome sequence phylogenetic tree shows the position of *B. bacteriovorus* YOA24 and YOA38 within the *B. bacteriovorus*. The phylogenetic tree was drawn on PATRIC using 100 single-copy genes. RAxML analyzed the concatenated, aligned protein and single-copy genes to draw a phylogenetic tree based on 100 rounds of rapid bootstrapping (15). The generated phylogenetic tree was visualized on the interactive tree of life (16). The tree is rooted at the midpoint.

On the whole-genome phylogenetic tree (Fig. 3), YOA24 and YOA38 were found in the *B. bacteriovorus* cluster, apart from other established *Bdellovibrio* species, *Bdellovibrio svalbardensis* and *Bdellovibrio reynosensis*, and *Bdellovibrio* sp. NC01, an established divergent *Bdellovibrio* strain (12–14). The whole-genome sequence phylogenetic tree established YOA24 and YOA38 as members of the *B. bacteriovorus* species.

### *B. bacteriovorus* YOA24 and YOA38 kill *Salmonella* using periplasmic invasion

**Fig 4:**
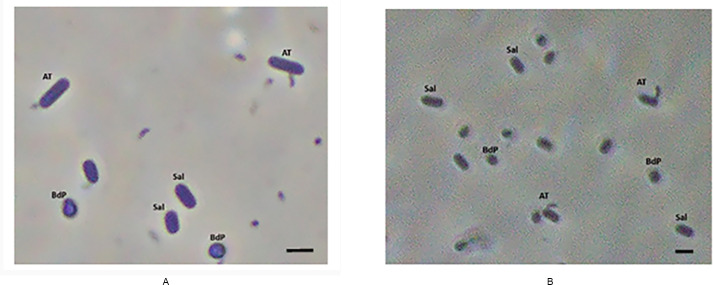
(**A**) A co-culture of *B. bacteriovorus* YOA24 and *S*. Infantis and (**B**) a co-culture of *B. bacteriovorus* YOA38 and *S*. Infantis. To view the culture, 50 µL of the co-culture containing *Salmonella* and the predatory bacteria (either strain YOA24 or YOA38) was placed on a slide with a cover slip on. The slide was observed under oil immersion at 1,000× magnification. *Salmonella* was observed by phase-contrast microscopy at 1,000× as rod-shaped bacteria, while *B. bacteriovorus* YOA24 or YOA38 was observed as comma shape attached to the rod-shaped bacteria. The infection of the rod-shaped *Salmonella* resulted in the presence of bdelloplast, a condition in which the rod-shaped bacteria are transformed into a spherical form. This microscopic observation establishes that *B. bacteriovorus* YOA24 and YOA38 show predation strategy typical of periplasmic *bdellovibrio*. Figure legend: AT, attachment of *Bdellovibrio* to *Salmonella*; Sal, *Salmonella*; BdP, bdelloplasts. The scale bar is 2 µm.

Under microscopic examination, YOA24 and YOA38 were observed to be tiny, comma-shaped bacteria. The predation strategy, observed under the phase-contrast microscope for YOA24 and YOA38, was periplasmic (Fig. 4). Strains YOA24 and YOA38 attached to *S*. Infantis FSIS32003110 pESI invaded the rod-shaped bacteria and transformed them into a bdelloplast (a condition wherein the rod-shaped bacteria are transformed into a rounded shape due to *Bdellovibrio* infection). The attachment of strains YOA24 or YOA38 to *S*. Infantis in a position similar to other *Bdellovibrio* and, more importantly, the evidence of prey bdelloplast confirmed that strains YOA24 and YOA38 are periplasmic *Bdellovibrio* strains.

### *B. bacteriovorus* YOA24 and YOA38 demonstrate lytic activity on 11 *Salmonella* serovars of public health concern

Prey range testing showed the activity of YOA24 and YOA38 strains against a panel of 11 clinically significant *Salmonella* serovars (Table 1).

**TABLE 1:** Details of the *Salmonella* serovars used in this study^*c*^

Strain name	Year	Source	Antibiotic profile	Antibiotic resistance genes	Reference
*S*. Derby	2006	Turkey	TET, FIS	*aac6-Iy*, *aph3''-Ia*, *aadA2, tetA*, *tetR*, *sul-I*, *fosA*	(17)
*S*. Enteritidis	2001	Chicken	ND	*aac6-Iy*	(17)
*S*. Hadar	2004	Environmental	STR, TET, FIS	*aac6-Iy*, *aph3''-Ia*, *strA*, *strB*, *aadA1-pm*, *tetA*, *tetR*, *sul-I*	(17)
*S*. Heidelberg	2004	Swine	AMP, GEN, STR, TET, FIS, COT	*aac6-Iy*, *aac-Iva*, *aphA2, sph, aph4-Ia*, *straA*, *strB*, *aadA2*, *tem*, *tetB*, *sulIII*, *fosA*, *drfA12*	(17)
*S*. IIIa 18:z4,z23:-		Unknown	GEN, STR, FIS	*aac3-Via*, *aadA1-pm*, *sul-I*	(17)
*S*. Infantis	2005	Chicken	AMP, FOX, TIO, AUG, AXO	*aac6-Iy*, *cmy-94*	(17)
*S*. Infantis FSIS12031643 pESI^*a*^	2020	Turkey	ND	*aac (3)-Iva*, *aph (4)-Ia*, *dfrA14, floR, mdsA*, *mdsB*, *gyrA_D87Y*	(18)
*S*. Infantis pESI FSIS32003110^*b*^	2019	Turkey	ND	*aac (3)-IVa*, *aadA1*, *aph (4)-Ia*, *blaCTX-M65*, *floR*, *fosA3*, *mdsA*, *mdsB*, *sul1*, *tet(A*), *gyrA_D87Y*	(18)
*S*. Javiana	2004	Dog	ND	*aac6-Iy*	(17)
*S*. Kentucky	2009	Chill water, poultry environment	STR, TET, FIS	*aac6-Iy*, *strA*, *strB*, *tetB*,	(17)
*S*. Thompson	2007	Chicken	TET	*aac6-Iy*	
*S*. Typhimurium	2007	Turkey	AMP, STR, TET, FIS, CHL	*aac6-Iaa*, *aph3''-Ia*, *strA*, *strB*, *aadB aadB*, *cmy60, tem-148 tetA terR*, *tetB*, *sul-II*, *floR*, *cmlA5*	(17)

^*a*^ *S*. Infantis pESI FSIS12031643 was used in the predation efficiency experiment.

^*b*^ *S*. Infantis pESI FSIS32003110 was used as the prey bait for isolating *B. bacteriovorus* strains 413 YOA24 and YOA38.

^*c*^ ND, not determined.

The results showed that YOA24 and YOA38 possess lytic activity on all 11 *Salmonella* serovars tested (Table 2). In a modified spot test, concentrations of *Bdellovibrio* ranging from 10^8^ to 10^1^ PFU/mL were tested on the same *Salmonella* prey lawn (see Fig. S1 for workflow and example image). Higher concentrations of both YOA24 and YOA38 (10^8^ to 10^5^ PFU/mL) produced clearances in the prey lawn within 24–72 h of incubation for each of three biological replicates, demonstrating these isolates’ ability to kill different *Salmonella* serovars. At 10^4^ PFU/mL and lower, YOA24 and YOA38 did not consistently produce noticeable clearances across replicates.

**TABLE 2:** Prey range of *B. bacteriovorus* YOA24 and *B. bacteriovorus* YOA38 by modified spot test^*a*^

	*B. bacteriovorus* YOA24							*B. bacteriovorus* YOA38						
	10^8^	10^7^	10^6^	10^5^	10^4^	10^3^	10^2^	10^8^	10^7^	10^6^	10^5^	10^4^	10^3^	10^2^
*S*. Derby	+++	+++	+++	+++	++	++	++	+++	+++	+++	+++	+++	++	++
*S*. Enteritidis	+++	+++	+++	+++	+++	+++	+++	+++	+++	+++	+++	+++	+++	+++
*S*. Hadar	+++	+++	+++	+++	+++	+++	+++	+++	+++	+++	+++	+++	+++	++
*S*. Heidelberg	+++	+++	+++	+++	+++	+++	+++	+++	+++	+++	+++	+++	+++	++
*S*. IIIa 18:z4,z23:-	+++	+++	+++	+++	+++	+++	+	+++	+++	+++	+++	+++	++	
*S*. Infantis	+++	+++	+++	+++	++	++	+	+++	+++	+++	+++	++	+	+
*S*. Infantis pESI FSIS12031643	+++	+++	+++	+++	+++	++	++	+++	+++	+++	+++	+++	+++	+
*S*. Javiana	+++	+++	+++	+++	+++	+++	++	+++	+++	+++	+++	+++	+++	+++
*S*. Kentucky	+++	+++	+++	+++	+++	+++	+++	+++	+++	+++	+++	+++	+++	+++
*S*. Thompson	+++	+++	+++	+++	+++	+++	+++	+++	+++	+++	+++	+++	++	++
*S*. Typhimurium	+++	+++	+++	+++	+++	+++	+	+++	+++	+++	+++	+++	+++	++

^*a*^ In the modified spot test, concentrations of YOA24 or YOA38 ranging from 10^8 ^to 10^1^ PFU/mL were tested against prey lawns of 11 *Salmonella *serovars. Spots were observed for clearances in the prey lawn during 24–72 h of incubation. Three biological replicates were performed for each *Bdellovibrio*-prey pairing. Clearance is represented with a + sign, with the number of positive signs (up to three) corresponding to the number of replicates for which clearance was observed.

### *B. bacteriovorus* YOA24 and YOA38 reduce *S*. Infantis pESI by 2 logs

**Fig 5:**
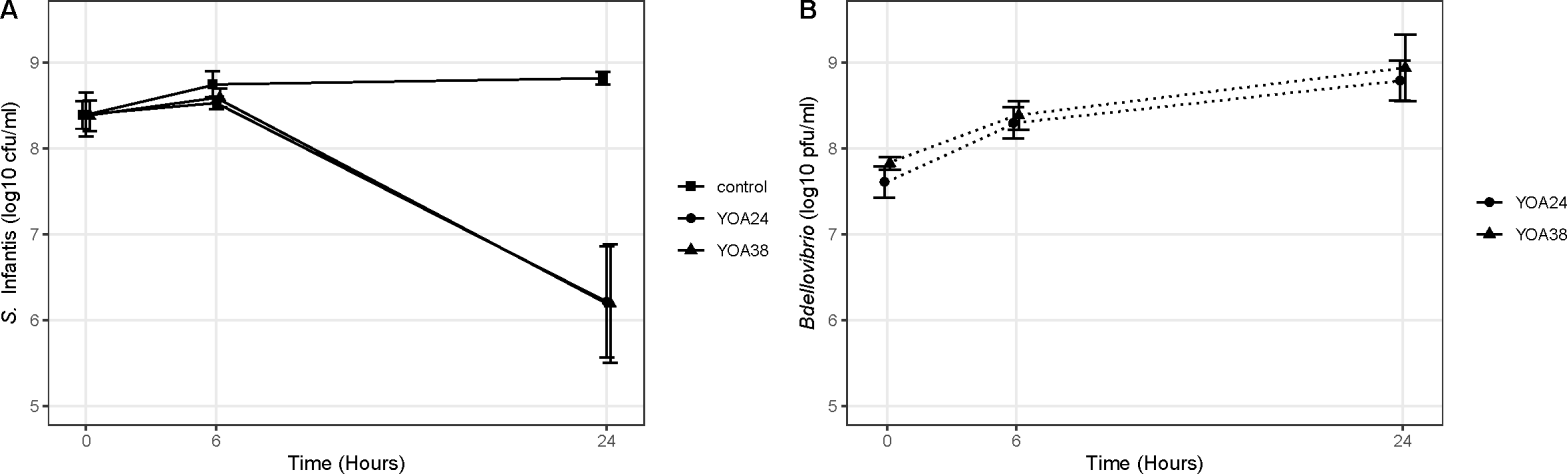
(**A**) Reduction in *S*. Infantis by *B. bacteriovorus* YOA24 and YOA38, while (**B**) shows an increase in YOA 24 and YOA38 over 24 h.

*B. bacteriovorus* YOA24 and YOA38 were screened for their ability to reduce *S*. Infantis in 24 h. *S*. Infantis FSIS12031643 pESI was reduced by 2 logs over 24 h while the population of *S*. Infantis FSIS12031643 pESI in the control remained constant. The reduction by 2 logs indicated that 99% of *S*. Infantis FSIS1203164 pESI have been removed in 24 h, making the strains YOA24 and YOA38 good disinfecting candidates. YOA24 and YOA38, reducing *S*. Infantis by 2 logs, performed similarly in the predation efficiency assay (Fig. 5A). While the prey population was observed to be reduced by 2 logs during the predation assay, the predators were observed to increase by at least a log (Fig. 5B). Thus, the killing of *Salmonella* increased the number of *Salmonella* killers (increase in the population of *B. bacteriovorus* YOA24 and YOA38).

## DISCUSSION

*Salmonella* is a significant foodborne pathogen responsible for over 1.3 million infections in the USA annually(19). Nontyphoidal *Salmonella* is a leading cause of illness, hospitalization, and even death among all foodborne bacterial pathogens (20). The CDC estimates that *Salmonella* causes more foodborne illnesses than other bacteria, with chicken as the primary source. *S. enterica* serovars are increasingly becoming a foodborne pathogen of significant public, economic, and health concern, with some strains linked to endemic, emerging, and reemerging diseases (20–22). The rise in resistance to established and novel antibiotics among *Salmonella* strains underscores the need for effective biological control methods, with predatory bacteria emerging as a promising candidate. *Bdellovibrio*, an obligatory predatory bacterium, has shown potential as both a probiotic and an antibiotic (4, 23). Its killing activity associated with its predatory lifestyle makes it a potent antimicrobial agent against gram-negative bacteria, including *Salmonella* (4, 24).

Despite the potential use of *Bdellovibrio* as a biological control of *Salmonella,* only a few strains have been tested. *B. bacteriovorus* HD100 was tested against *S. enterica in vitro* and *in vivo* (24). It was reported that 97% of *S. enterica* serovar Enteritidis was removed in a liquid assay within 24 h, while a log reduction was observed when applied *in vivo* in chickens (24). *B. bacteriovorus* 109J was tested on *S. enterica* serovars (*S*. Arizonae, *S*. Dublin, *S*. Enteritidis, *S*. Seftenberg, *S*. Poona, and *S*. Typhimurium) (25). It was observed to be effective on *S*. Poona only, suggesting that the prey range and efficiency of *Bdellovibrio* can vary across different *Salmonella* serovars. The need to screen for potent predatory bacteria against a wide range of *Salmonella* serovars of public health, safety, and economic concern requires screening for efficient *Bdellovibrio*. In this study, two *B. bacteriovorus* strains were isolated from natural environments using *S*. Infantis as the prey bait; the strains demonstrated killing activity on all *Salmonella* serovars tested, thus indicating their potential to be developed into an intervention for the control of *Salmonella*.

The two *B. bacteriovorus* strains, YOA24 and YOA38, isolated from the Upper Oconee River Watershed, support the hypothesis that where the prey is, there is the predator. Our previous study recovered *Salmonella* from the two sampling sites at least twice within four seasons (winter, spring, summer, and fall) between 2015 and 2017 (26). This current study establishes the presence of *Bdellovibrio* in the natural aquatic environment (an urban watershed), making it another *B. bacteriovorus* strain reported to be isolated from water. Their isolation confirms the ubiquitous nature of *Bdellovibrio. B. bacteriovorus* Tiberius was isolated from the Tiber River, while *B. bacteriovorus* HD100, 109J, and SSB218315 were isolated from soil (27–29). *Bdellovibrio* has been isolated from freshwater, seawater, soil, rhizospheres, sewage, wastewater sludge, and human and animal intestines (30). Despite recovering *B. bacteriovorus* from the two sampling sites, we cannot conclude that these strains are the most dominant predatory bacteria at each site. The diversity of *Bdellovibrio* species at each site was not quantified. With the sampling size and technique (which involved picking a single plaque as a representative strain from each site) used, it would be biased to assert that *B. bacteriovorus* is the dominant strain. Nonetheless, *B. bacteriovorus* remains the most extensively studied among all obligatory predatory bacteria.

Previous *Bdellovibrio* prey range assessments have focused on prey of diverse phylogenetic genera, with a limited study testing predatory activity on members of the same species. The 11 serovars tested are serovars of *S. enterica* associated with beef, pork, poultry, or seafood and can be transmitted to humans via the food chain, causing gastroenteritis and diarrhea in humans (31). The newly isolated strains *B. bacteriovorus* YOA24 and YOA38 could have a broad prey range for diverse *Salmonella* serotypes. Oyedara et al. (28) reported the ability of *B. bacteriovorus* SSB218315 or *Bdellovibrio* sp. SKB1291214 isolated from soil to prey on all four *Salmonella* serotypes tested: *Salmonella enterica* subsp. *enterica* serovar Typhi CDBB-B-1101 (ATCC 7251), *Salmonella* sp. A, *Salmonella* sp. B, and *Salmonella* sp. D (28). *B. bacteriovorus* 109J could prey only on *S*. Poona after 7 h of incubation, but not on *S*. Enteritidis Y8P2. At various culture conditions, such as pH and temperature, *B. bacteriovorus* 109J could not prey on *S*. Enteritidis Y8P2 (25).

*Bdellovibrio* strains exhibit prey preference, but the genetic mechanisms underlying these variations are not fully understood (14, 32). *B. bacteriovorus* 109J was reported to show differential predation, killing some prey more readily than others (33). It was reported that *B. bacteriovorus* 109J preferred *Pantoea agglomerans,* followed by *Serratia marcescens,* as prey over *Escherichia coli, Enterobacter aerogenes,* and *Erwinia carotovora* subsp. *carotovora*, or *S. enterica* (33), though it killed all six of the prey. The differences in the *Bdellovibrio*-prey predation are a product of the *Bdellovibrio*-prey interaction. Prey’s susceptibility to *Bdellovibrio* predation depends on several factors, including the outer membrane of the prey surface. Factors responsible for the predator-prey interaction are poorly understood and have been described as multifactorial (34). *Bdellovibrio’s* inability to kill its prey or the prey’s ability to escape predation in some conditions has been reported (6). However, this is not within the scope of this study. We conclude that *Bdellovibrio* killing activity against members of the same prey species could differ.

Obligate bacteria predators of gram-negative bacteria were previously classified based on the phenotypic profile of their prey range and described as *Bdellovibrio* and -like organisms (29). The classification of members of this group and their phylogenetic relatedness is better understood by using microbial genomics tools. The 16S rRNA gene, *rpoB* gene, GC content, DNA-DNA hybridization, and whole-genome analysis are helping to classify bacteria that would have been previously grouped as *Bdellovibrio* and -like organisms. All *B. bacteriovorus* strains, HD100, 109J, SSB218315, Tiberius, YOA24, and YOA38, have a 50%–50.5% GC content. With the availability of whole-genome sequencing, DNA-DNA hybridization has been replaced with dDDH, which can be determined by submitting genomes for dDDH calculation. Members of the same species are identified to have a >70% dDDH value. The 16S rRNA sequencing, GC contents, dDDH values (>70%), and whole-genome phylogenetic tree established the newly isolated strains YOA24 and YOA38 as members of the *B. bacteriovorus* species. *B. bacteriovorus* YOA24 shares a closer relationship with HD100 and 109J, strains isolated from soil samples in California, USA, while YOA38 shares a closer relationship with SSB218315, a strain isolated from Reynosa, Mexico. It is doubtful if geographical location (these strains have been recovered from North America) contributed to the relatedness of the strains, as *B. bacteriovorus* Tiberius, a strain within the same clade, was isolated from the River Tiber in Rome.

Predation by obligate predatory bacteria has been reported to be periplasmic or epibiotic. Our microscopic observation supports the conclusion that strains YOA24 and YOA38 are periplasmic predators like the well-characterized *B. bacteriovorus* HD100 and 109J. *B. bacteriovorus* YOA24 and YOA38 have a GC content genome size of 50% and 50.5%, 3.9 MB each, and periplasmic predators have GC content ranging from 43 to 51%, while sequenced epibiotic predators have less than 43% GC content. Reported periplasmic *Bdellovibrio*, *B. bacteriovorus* HD100, 109J, SSB2181315, and Tiberius, *B. reynosensis*, *B. svalbardensis,* and *Bdellovibrio* sp. NC01, have genome sizes within the 3.6 and 4 MB range, while the epibiotic predator *Bdellovibrio* sp. Qaytius and *Pseudobdellovibrio exovorus* have genome sizes of 3.3 MB and 2.7 MB, respectively. Our microscopic observations established strains YOA24 and YOA38 as periplasmic predators, which is supported by the phylogenetic placement. The phylogenetic tree places YOA24 and YOA38 within the established periplasmic *Bdellovibrio* and away from the epibiotic *Bdellovibrio sp*. Qaytius or *Pseudobdellovibrio exovorus*. Strains YOA24 and YOA38 are periplasmic predatory bacteria within the group *B. bacteriovorus*.

This study isolated two *Bdellovibrio bacteriovorus* strains, *B. bacteriovorus* YOA24 and YOA38, from a natural environment. The strains were identified as periplasmic *Bdellovibrio* and members of the *B. bacteriovorus*. These newly isolated strains indicate their potential for managing *Salmonella* outbreaks. They have demonstrated the ability to predate on 11 *Salmonell*a serovars and effectively reduce *S*. Infantis populations. These *Bdellovibrio* strains may be developed as probiotics to control foodborne *Salmonella*.

## MATERIALS AND METHODS

### *Salmonella* strains

*Salmonella* (*n* = 12) used as prey in this study were previously isolated from food animals between 2001 and 2020 (17, 18). These strains represent the major foodborne-associated *Salmonella* serovars that cause human illness (Table 1).

### Isolation of *B. bacteriovorus* strains YOA24 and YOA38

YOA24 and YOA38 were isolated from the Upper Oconee Watershed in Athens, Georgia, at locations 33.92973 N, −83.456502 W, and 33.957 N, −83.3665 W, respectively, using a modified technique of Jurkevitch (35) as described below. YOA24 and YOA38 were isolated using *S*. Infantis FSIS32003110 pESI as prey bait.

A sterile swab was dipped into the water to isolate naturally occurring *Bdellovibrio* from the two sampling sites, or a sterile swab was rubbed on a solid surface submerged at the shoreline. The swabs (*n* = 6 per site) were stored in 20 mL sterile HM buffer (25 mM HEPES adjusted to pH 7.2 and supplemented with 3  mM calcium chloride dihydrate and 2 mM magnesium chloride hexahydrate) in a 50 mL sterile Falcon tube and taken to the laboratory for further processing. The tubes were placed in an incubator shaker at 30°C for 1 h to dislodge the collected sample into the HM buffer, which was then filtered through a 1.2 µm, 0.8 µm, and 0.45 µm filter. Ten milliliters of filtrate was then combined with *S*. Infantis to enrich for *Bdellovibrio*. To obtain an overnight culture of *Salmonella* for use as prey in enrichment, a single colony of *S*. Infantis was picked from a blood agar plate, inoculated into 20 mL tryptic soy broth, and incubated at 36°C for approximately 18 h. After centrifugation at 12,857 *g* for 20 min, *S*. Infantis cells were collected and washed twice in HM buffer. The resulting cell pellet was resuspended in 20 mL sterile HM buffer, then combined with 10 mL of the filtrate and 20 mL of sterile HM buffer, and then placed in an incubator shaker at 30°C for 72 h.

During incubation, samples were observed daily using 1,000× phase-contrast microscopy under oil immersion for tiny fast-moving bacteria typical of *Bdellovibrio* or evidence of *Bdellovibrio* attachment to prey. Samples showing evidence of bacteria cells typical of *Bdellovibrio* were serially diluted by a 10-fold serial dilution in HM buffer by removing 100 µL of culture into 900 µL fresh HM buffer and continuing the dilution to 10^6^. Diluents were then plated using the double agar overlay technique (35). Briefly, 100 µL of the diluent was combined with 300 µL of *S*. Infantis (1 × 10^9^ CFU/mL) in 7 mL molten 0.6% HM top agar, vortexed quickly, and poured onto 18 mL bottom 1.5% HM agar. The plates were allowed to sit undisturbed at room temperature before incubating at 30°C and were observed daily for plaque formation (plaque was first observed as a small circle formed on the prey’s lawn). Evidence of plaques within 2–5 days suggested the presence of *Bdellovibrio*. During the isolation, we made use of HM media, as this has no nutrients at all and it will not allow the growth of other bacteria from the environment, which might pass through 0.45 µm filter to compete with *Bdellovibrio*.

The isolate was observed as plaques and purified thrice by cutting a single plaque per sampling site to repeat the double agar overlay technique to obtain pure strains from a single plaque (14). To achieve this, a single well-separated plaque was removed from the plate using a sterile borer into 600 µL HM buffer and vortexed vigorously before incubating overnight at 30°C. Aliquots were taken for observation under the phase-contrast microscope before continuing with another round of double agar overlay assay. The process was repeated using the same prey to obtain pure isolates from a single plaque. After the third round, a single plaque was cut out and placed into 5 mL HM buffer with an overnight culture of *S*. Infantis and incubated at 30°C for 3 days. The culture was observed under the microscope to contain *Bdellovibrio* and then filtered with a 0.45 µm filter. The filtrate was stored in 50% glycerol at −80°C (14).

### Preparation of *B. bacteriovorus* YOA24 and YOA38 for microscopic observation

To obtain a microscopic view of the strains using phase-contrast microscopy, stocks of YOA24 and YOA38 were revived from −80°C by adding 10 µL of the glycerol stock and 1.5 mL overnight culture of *S*. Infantis pESI FSIS32003110 into 20 mL HM buffer. The culture was placed in an incubator shaker set at 30°C. The lysate was then prepared for microscopic observation (14, 36).

### Prey range and predation efficiency of strains YOA24 and YOA38

The ability of strains YOA24 and YOA38 to prey on important antibiotic-resistant *Salmonella* serovars (Table 1) was investigated using a modified spot test technique (37). Stocks of YOA24 and YOA38 were revived from −80°C by adding 10 µL of the glycerol stock and 1.5 mL of an overnight *E. coli* ML35 culture into 20 mL sterile HM buffer. *E. coli* ML35 was chosen as it is common prey used in *Bdellovibrio* research and to avoid priming the *Bdellovibrio*. The culture was placed in an incubator shaker at 30°C for 48 h. The lysate was then filtered through a 0.45 µm filter, and the filtrate was adjusted to contain *Bdellovibrio* concentration of approximately 1–5 × 10^8^ PFU/mL based on a microscopic view and confirmed by plaque counts on a double-layer diluted nutrient broth (DNB) agar plate using *E. coli* ML35 as the prey.

A 10-fold serial dilution of the 10^8^ PFU/mL *Bdellovibrio* was carried out, from which 10 µL of each dilution was spotted on a lawn of the *Salmonella* strains under testing (the *Salmonella* strains under testing were revived by taking out the stock culture stored in glycerol at −80°C and streaking a loopful on a blood agar plate). To make a lawn of *Salmonella* on a DNB agar plate, a single colony of the *Salmonella* strain from the blood agar was inoculated into Luria-Bertani (LB) broth and incubated overnight at 36°C. The broth culture was adjusted to an OD_600_ nm of 0.2–0.3, from which 500 µL was placed into 7 mL DNB top agar, vortexed rapidly, and poured onto an 18 mL DNB bottom agar plate. We made use of DNB during the spot test as it contains a little nutrient that allows for *Salmonella* to grow, thus giving us a visible lawn wherein the activity of the *Bdellovibrio* can be easily noticed. The HM and DNB agar are both agars known for culturing *Bdellovibrio* (36). The plates were incubated at 30°C and observed daily for clearance zones. Clearance between 24 and 72 h of incubation was scored as positive (+) (Fig. S1). Experiments were performed as three biological independent replicates.

The predation efficiency tests were performed in a co-culture assay. *S*. Infantis at a minimum of 1 × 10^8^ CFU/mL concentration was combined with either YOA24 or YOA38 at a minimum of 1 × 10^7^ PFU/mL in HM buffer. A control with *S*. Infantis pESI FSIS12031643 only, and no *Bdellovibrio,* was set up alongside. To test for predation efficiency, a single colony of *S*. Infantis FSIS12031643 pESI on a blood agar plate was inoculated in LB broth to obtain an overnight culture with a CFU/mL of approximately 10^8^. The *Bdellovibrio* strains, adjusted to approximately 10^7^ PFU/mL concentration, were obtained as stated above, as in the prey range testing. The predator strain (2 mL) and *S*. Infantis (2 mL) were inoculated into 20 mL HM to obtain an average predator:prey ratio of approximately 2:10 for YOA24 and 3:10 for YOA38. The experiment was conducted in an incubator shaker at 30°C for 24 h. The culture was shaken for 24 h, and a 100 µL sample was withdrawn from the culture flasks at 0, 6 h, and 24 h. During this period (0, 6 h, and 24 h), the sample (100 µL) removed from the flask was diluted (10-fold serial dilution) to quantify *Bdellovibrio* and *Salmonella*. Samples withdrawn at 0, 6 h, and 24 h were quantified for *S*. Infantis pESI FSIS12031643 in CFU/mL using the spread plate technique and *Bdellovibrio* in PFU/mL using the double-layer overlay agar technique. For the predator to prey ratio, while the prey could be estimated by OD_600_ corresponding to a CFU/mL, the predator could not be estimated in such a manner. Instead, the predator concentration was adjusted by microscopic observations, and then the PFU/mL was quantified by plating with prey in a double-layer plaque assay. It then takes approximately 3 days for the plaques to form and be counted to determine the actual PFU/mL. Therefore, the exact amount of predator can only be determined 3 days after the experiment is over, and that number is used to calculate the predator:prey ratio, and this is responsible for the differences in ratios. Despite this limitation, Fig. 5 suggests that these initial concentrations do not show a significant difference.

### The whole-genome sequencing, assembly, and annotation

Strains YOA24 and YOA38 were revived from −80°C as described above. Their co-culture was filtered through a 0.45 µm filter to remove residual prey. The prey-free filtrate was concentrated by centrifugation at 12,857 *g* at 4°C for 30 min. The cells were collected for DNA extraction using the Wizard genomic DNA purification Kit (Promega, Madison, WI, USA), following the manufacturer’s instructions. The precipitated DNA was rehydrated in 1× tris EDTA buffer (pH 8.0) and stored at −20°C. The extracted genomic DNA was quantified using a Nanodrop spectrophotometer and a Qubit 2.0 Fluorometer (Thermo Fisher Scientific, Waltham, MA, USA) using the Qubit double-stranded DNA high-sensitivity kit, following the manufacturer’s instructions (Life Technologies Inc., Carlsbad, CA, USA). The extracted DNA was used for genome sequencing.

The Nextera XT DNA Sample Preparation and a Nextera XT Index Kit (Illumina Inc., San Diego, CA, USA) were used to prepare genomic libraries. The libraries were quantified using a Qubit Fluorometer, while the size of the fragment libraries was determined on a Bioanalyzer 2100 using an Agilent High Sensitivity DNA Kit (Agilent Technologies, Santa Clara, CA, USA). The libraries were pooled and sequenced on an Illumina MiSeq platform (Illumina Inc., San Diego, CA, USA) using a MiSeq v2 reagent kit with 500 cycles and a paired-end read length of 2 × 250 bp. The Illumina MiSeq sequencing reads were checked for quality using FastQC, low-quality reads were removed using Trimmomatic, and reads were assembled *de novo* using the A5-miseq assembler. The shotgun Illumina reads have been deposited in the Sequence Read Archive (SRA) database under the BioProject accession number PRJNA1226776 for *B. bacteriovorus* YOA24 and YOA38. The assembled draft genome in contigs is deposited in the National Center for Biotechnology Information (NCBI) GenBank as GCF_048286975.1 and GCF_048286055.1 for *B. bacteriovorus* YOA24 and YOA38, respectively.

### Identification and phylogenetic relationship of strains YOA24 and YOA38 with other *Bdellovibrio* strains

To identify strains YOA24 and YOA38, a BLAST (Basic Local Alignment Search Tool) of the 16S rRNA gene sequences (1,514 bp) extracted from their genomes was made on the rRNA_typestrains/16S_ribosomal_RNA NCBI BLAST and the EZbiocloud 16S-based ID database. The NCBI BLAST results further the need to identify the relationship between strains YOA24, YOA38, and *B. bacteriovorus* HD100. Thus, the dDDH calculator was used to investigate the relationship between these strains (38). The evolutionary relationship of the two newly isolated *Bdellovibrio* strains to other members of the *Bdellovibrio* was inferred via a phylogenetic tree. The whole-genome sequence tree was drawn on PATRIC (15). PATRIC allows the user to specify between 10 and 1,000 protein families that are single copies. RAxML analyzed the concatenated, aligned protein and single-copy genes to draw a phylogenetic tree based on 100 rounds of rapid bootstrapping. A phylogenetic tree was generated using 100 single-copy genes (15). The generated phylogenetic tree was visualized on the interactive tree of life (16).

## Data Availability

The shotgun Illumina reads have been deposited in the Sequence Read Archive (SRA) database under the BioProject accession number PRJNA1226776 for *B. bacteriovorus* YOA24 and YOA38. The assembled draft genome in contigs is deposited in the National Center for Biotechnology Information (NCBI) GenBank as GCF_048286975.1 and GCF_048286055.1 for *B. bacteriovorus* YOA24 and YOA38, respectively.

## SUPPLEMENTAL MATERIAL

Fig. S1 spectrum.00861-25-s0001.tif Description of the spot test used in prey-range testing.

Supplemental legend spectrum.00861-25-s0002.docx Legend for Fig. S1.

Table S1 spectrum.00861-25-s0003.xlsx Genome statistics after the Illumina reads were assembled using the A5-miseq assembler.

ASM does not own the copyrights to Supplemental Material that may be linked to, or accessed through, an article. The authors have granted ASM a non-exclusive, world-wide license to publish the Supplemental Material files. Please contact the corresponding author directly for reuse.
