# Supplementary figures and images for: Antibacterial activity of two newly isolated Bdellovibrio bacteriovorus strains on Salmonella enterica serovars of food safety concern
Source: Microbiol Spectr. 2025 Sep 15;13(10):e00861-25. doi: 10.1128/spectrum.00861-25 (PMC12502522; doi:10.1128/spectrum.00861-25)

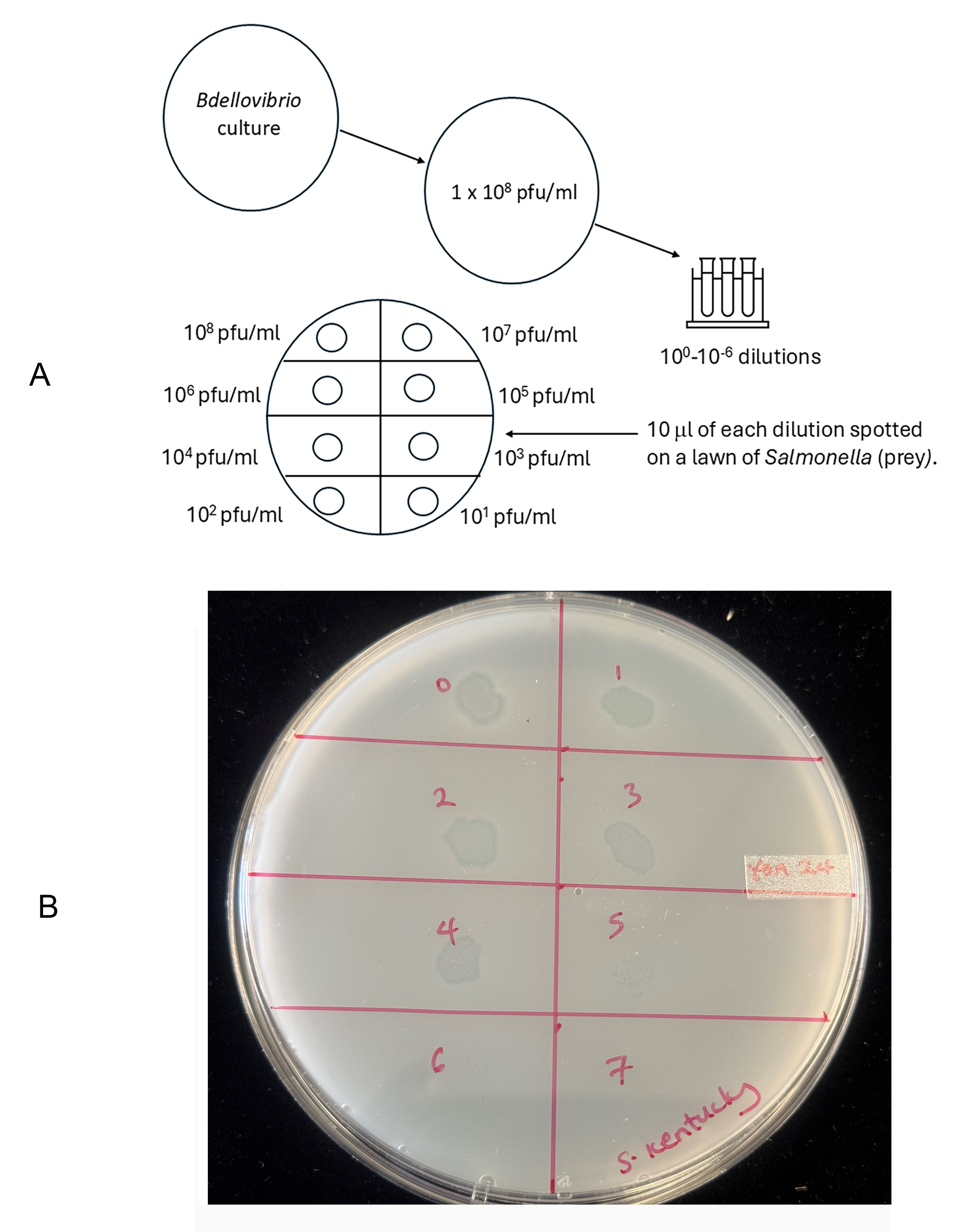

Supplement: Fig. S1 — Description of the spot test used in prey-range testing. [file spectrum.00861-25-s0001.tif]
